# First Report from Colombia of a Urinary Tract Infection Caused by *Kluyvera ascorbata* Exhibiting an AmpC Resistance Pattern: A Case Report

**DOI:** 10.3390/diseases13070194

**Published:** 2025-06-25

**Authors:** Esteban Artunduaga-Cañas, Sinthia Vidal-Cañas, Valentina Pérez-Garay, Johnny Valencia-Ibarguen, Diego Fernando Lopez-Muñoz, Yamil Liscano

**Affiliations:** Grupo de Investigación en Salud Integral (GISI), Departamento Facultad de Salud, Universidad Santiago de Cali, Cali 760035, Colombia; esteban.artunduaga01@uceva.edu.co (E.A.-C.); sinthia.vidal00@usc.edu.co (S.V.-C.); valentina.perez01@usc.edu.co (V.P.-G.); jhonny.valencia00@usc.edu.co (J.V.-I.); dflopez@uceva.edu.co (D.F.L.-M.)

**Keywords:** case report, urinary tract infection, *Kluyvera ascorbata*, antibiotic, *Enterobacteriaceae*, beta-lactam resistance

## Abstract

**Background**: Urinary tract infections represent a significant healthcare burden, particularly among vulnerable patients with chronic comorbidities. In this case report, we describe a UTI caused by *Kluyvera ascorbata* exhibiting an *AmpC* resistance pattern in an 85-year-old male with stage IV chronic kidney disease and a history of ESBL-positive infection. **Methods**: A comprehensive diagnostic workup was performed, including clinical evaluation, laboratory tests (urinalysis, complete blood count, renal function tests), and microbiological cultures with antibiogram analysis using the MicroScan WalkAway (Beckman Coulter, Brea, United States) and VITEK2 Compact systems (bioMérieux, Marcy L’Étoile or Craponne, France). **Results**: The initial urine culture revealed a Gram-negative bacillus and subsequent identification confirmed *K. ascorbata*, which demonstrated resistance to ampicillin and cefazolin while remaining susceptible to meropenem. The patient received intravenous meropenem therapy for 10 days, resulting in clinical improvement and a subsequent negative urine culture. **Conclusions**: This case reports a complicated urinary tract infection caused by *K. ascorbata* with an AmpC resistance pattern, highlighting the importance of considering this infrequently reported pathogen and its resistance profile in vulnerable patients. Its multidrug-resistant profile underscores the necessity for vigilant antimicrobial stewardship and further research to develop standardized treatment protocols for managing infections caused by this organism.

## 1. Introduction

Urinary tract infections (UTIs) represent a significant global health burden, being among the most frequent community-acquired and healthcare-associated infections. They are a leading cause of bacteremia and sepsis, particularly in older adults and individuals with underlying comorbidities [1,2,3,4]. The risk of UTIs increases considerably in vulnerable populations, including men over 50, for whom it is often linked to conditions like prostatic hyperplasia, and patients with indwelling urinary catheters, which impair bladder emptying and promote bacterial colonization [5,6,7].

*Kluyvera* species, historically considered primarily environmental organisms, are now recognized as opportunistic pathogens capable of causing a range of clinically significant infections in humans, including UTIs, sepsis, and intra-abdominal abscesses [1,8,9]. Belonging to the *Enterobacteriaceae* family, *Kluyvera* spp. are of increasing clinical concern due to their notable capacity to acquire and disseminate antimicrobial resistance genes. These include genes encoding extended-spectrum β-lactamases (such as *CTX-M* enzymes, sometimes associated with *bla*KLUA), carbapenemases (like *blaNDM-1*)*,* and even colistin resistance determinants (*mcr-1*), posing significant therapeutic challenges, especially in multidrug-resistant strains [10,11,12,13,14,15,16].

Despite their growing clinical relevance and concerning resistance profiles, *Kluyvera* infections remain underrecognized, and reports from certain regions, including Latin America, are scarce. To our knowledge, we present the first documented case in Colombia of a complicated urinary tract infection caused by *Kluyvera ascorbata* exhibiting a resistance profile suggestive of AmpC β-lactamase activity. This report highlights the importance of considering this pathogen in vulnerable patients with complex comorbidities and underscores the need for enhanced regional surveillance and microbiological diagnostic capacity.

In the following sections, we detail the clinical presentation, microbiological findings, and therapeutic management of this case, providing valuable insights into *K. ascorbata* infections in a high-complexity hospital setting.

## 2. Case Presentation

**Figure 1:**
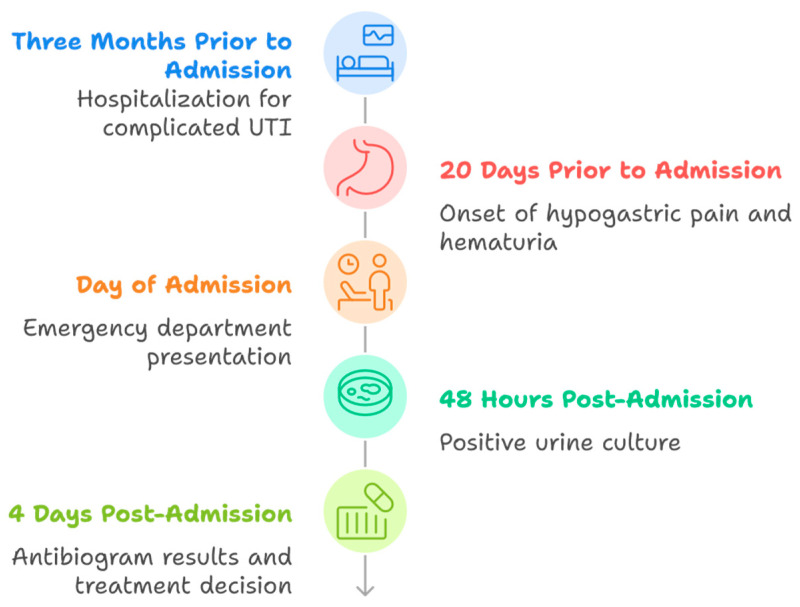
Timeline of clinical events.

Figure 1 illustrates the key chronological milestones in the patient’s clinical course, starting three months prior to admission with a complicated urinary tract infection that required hospitalization. Twenty days before admission, the patient developed hypogastric pain and hematuria, prompting an eventual visit to the emergency department on the day of admission. Forty-eight hours after admission, the initial urine culture confirmed the presence of a Gram-negative bacillus. Four days post-admission, the final antibiogram identified *K. ascorbata*, guiding the decision to maintain meropenem therapy due to the patient’s history of a recent ESBL-positive infection.

### 2.1. Initial Presentation and Medical History

An 85-year-old man with a history of benign prostatic hyperplasia (post-prostatectomy) and stage IV chronic kidney disease (GFR 26 mL/min, secondary to obstructive uropathy) presented with a 20-day history of hypogastric pain, obstructive urinary symptoms, and hematuria. Three months prior, he had been hospitalized for a urinary tract infection complicated by abnormal urinalysis findings. Initially treated with ceftriaxone, his antibiotic regimen was escalated to meropenem after urine culture revealed ESBL-positive, multidrug-resistant *E. coli*. Concurrently, the patient was under urological surveillance for suspected bladder cancer; a Urotac study had demonstrated a thick-walled, irregular bladder with an infiltrative, neoplastic appearance, and imaging revealed a metastatic pathological fracture at the T12 vertebral body.

### 2.2. Clinical Examination and Laboratory Evaluation

Upon admission, the patient was afebrile with stable vital signs. Physical examination revealed tenderness upon deep palpation of the hypogastric region without any palpable masses or signs of peritoneal irritation. A bladder catheter was in place, draining 300 mL with noted pyuria, and grade 1 pressure ulcers were observed in the sacral region. Neurologically, he was alert and oriented, with no motor or sensory deficits.

A comprehensive laboratory workup, including complete blood count, C-reactive protein (CRP), electrolytes, renal function tests, urinalysis, and urine culture, was performed (see Table 1). The urinalysis showed a cloudy yellow specimen with marked leukocyte esterase (+++), a blood level of 200, a negative bilirubin test, and a sediment containing 0–2 cells/xc, 40–60 red blood cells/xc, abundant bacteria, and uncountable leukocytes with 10% dysmorphic and 90% eumorphic cells. These findings, combined with the patient’s symptoms, confirmed a diagnosis of catheter-associated urinary tract infection with a Tumbarello score of 9.

### 2.3. Hospital Course and Management

The patient was admitted for inpatient care. He received intravenous fluids (0.9% normal saline at 20 mL per hour) and glycine solution for bladder irrigation. Intravenous meropenem (1 g every 12 h, adjusted for renal function) was administered for 10 days, along with omeprazole 20 mg taken on an empty stomach.

**Figure 2:**
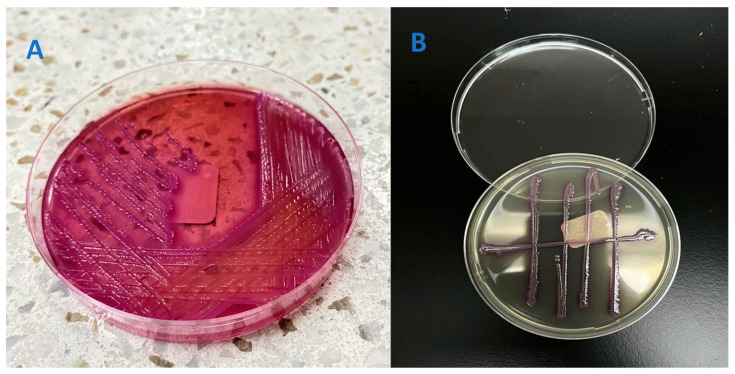
Isolation of a Gram-negative fermenting bacillus on MacConkey agar, showing (**A**) pinkish-red colonies characteristic of lactose fermentation and (**B**) a count higher than 100,000 CFU of a Gram-negative fermenting bacillus, interpreted as such on the MacConkey agar.

At 48 h, the urine culture was positive for a Gram-negative bacillus (Figure 2A). Four days later, the culture with an antibiogram identified *K. ascorbata* at a concentration exceeding 1,000,000 CFU/mL (Figure 2B). Despite considering de-escalation from carbapenems to a fourth-generation cephalosporin, the decision was made to continue the established meropenem therapy, given the recent history of a complicated ESBL-positive urinary tract infection.

### 2.4. Antibiotic Susceptibility Profile

Table 2 presents the antibiotic susceptibility profile obtained from two urine cultures (A and B) for *K. ascorbata*, isolated at a concentration of 100,000 CFU/mL. Two identification and susceptibility methods were employed (MicroScan WalkAway (Beckman Coulter, Brea, United States)) and VITEK2 Compact (bioMérieux, Marcy L’Étoile or Craponne, France). In both instances, the strain exhibited susceptibility to key antibiotics, including amikacin, cefepime, ertapenem, gentamicin, imipenem, and meropenem. However, discrepancies were observed for other agents: resistance to ampicillin and cefazolin was noted, and variable responses were seen for ampicillin–sulbactam and ciprofloxacin. The observed susceptibility pattern, particularly the resistance to ampicillin, cefazolin, and cefoxitin, along with the variable susceptibility to ampicillin–sulbactam and resistance to ceftriaxone ascertained by one method, is highly suggestive of acquired AmpC beta-lactamase production. AmpC enzymes confer resistance to penicillins, early-generation cephalosporins, cefoxitin, and often oxyimino-cephalosporins (like ceftriaxone, cefotaxime, and cefepime depending on the level of expression) and are typically not inhibited by beta-lactamase inhibitors such as sulbactam or clavulanic acid. The observed intermediate/sensitive results for ampicillin–sulbactam are consistent with incomplete inhibition of an AmpC enzyme by sulbactam. Susceptibility to carbapenems (ertapenem, imipenem, meropenem) further supports this phenotypic interpretation, excluding the presence of carbapenemases.

These findings underscore the importance of considering the testing method in the interpretation of the results and highlight the need for a comprehensive clinical evaluation to guide antibiotic management.

After medical intervention, the patient showed acceptable general conditions, with improvement in urine appearance and favorable response to antibiotics after finishing therapy; a second urine culture was confirmed with a negative result for the bacterium *K. ascorbata*. Clinical conditions and behaviors to be followed were explained to the patient, such as a healthy diet and increased physical activity.

## 3. Discussion

### 3.1. Main Findings and Comparative Analysis

This case highlights several key findings: An 85-year-old man with chronic kidney disease and a prior ESBL-positive infection developed a complicated, catheter-associated UTI. The initial urine culture revealed a Gram-negative bacillus, and subsequent identification confirmed *K. ascorbata* at a high concentration. Despite the possibility of de-escalating therapy, the decision to continue meropenem was made based on the patient’s history of resistant infections. These findings underscore the clinical significance of *K. ascorbata* as an emerging opportunistic pathogen, particularly in vulnerable patients with chronic comorbidities.

*K. ascorbata* has been historically underestimated, but its pathogenic potential is now well recognized. Although originally described in 1936 as a benign organism, subsequent studies have established its role in various infections, including UTIs, sepsis, intra-abdominal abscesses, and bacteremia [8]. Its ubiquity in environmental sources, ranging from water and food to hospital settings, combined with its ability to colonize the respiratory and gastrointestinal tracts, renders it particularly dangerous for immunocompromised individuals. In our patient, underlying conditions such as chronic kidney disease and previous infections further increased susceptibility.

The antimicrobial resistance profile of *K. ascorbata* is particularly concerning. The pathogen’s potential to harbor resistance genes, including *mcr-1* and *bla NDM-1* [9], poses significant therapeutic challenges. In our case, although the isolate remained susceptible to key antibiotics such as cefepime, ertapenem, gentamicin, imipenem, and meropenem, resistance to agents like ampicillin and cefazolin was evident. The variability observed in susceptibility to combinations such as ampicillin–sulbactam and ciprofloxacin emphasizes the need for careful interpretation of antibiograms and supports the continued use of broad-spectrum agents in patients with a history of multidrug-resistant infections.

Comparative studies have demonstrated that *K. ascorbata* infections are increasingly reported in patients with significant comorbidities, including those undergoing hemodialysis or with underlying malignancies. These studies, summarized in Table 3, underline the pathogen’s capability to cause severe infections such as sepsis and septic shock in vulnerable populations. Such findings justify the aggressive management approach taken in our case and highlight the importance of considering *K. ascorbata* in the differential diagnosis of complicated UTIs.

Drawing upon the recent literature, our isolate’s phenotypic profile, particularly the resistance to ampicillin, cefazolin, and cefoxitin, along with variable susceptibility to ampicillin–sulbactam and ceftriaxone, aligns with patterns suggestive of AmpC β-lactamase activity, a mechanism well described in *Enterobacteriaceae*. While *Kluyvera* species are known to carry chromosomal genes related to CTX-M-type ESBLs (*bla*KLUA), they typically lack a chromosomal *bla*AmpC gene, suggesting that the AmpC phenotype in our case is likely mediated by an acquired plasmid-borne enzyme. This contrasts with the intrinsic, inducible AmpC found in species like the *Enterobacter cloacae* complex or *Citrobacter freundii*, which are classified among *Enterobacterales* with a moderate-to-high risk of derepression upon beta-lactam exposure. Recent reports highlight challenges in accurately identifying *Kluyvera* and its resistance mechanisms, noting discrepancies between molecular methods and phenotypic susceptibility testing and demonstrating that some *Kluyvera* isolates can carry *bla*CTX-M genes yet remain susceptible to third- and fourth-generation cephalosporins. This underscores the complexity of resistance in *Kluyvera* and the critical need for reliable phenotypic testing, as performed in our case, to guide therapy. The decision to continue meropenem was also informed by the patient’s history and the need for reliable coverage against a potentially complex resistance profile, a strategy supported for severe infections involving multidrug-resistant *Enterobacterales* [17,18,19].

### 3.2. Clinical Implications

The clinical implications of this case are considerable, particularly in the context of regions like Colombia and Latin America, where reports of *Kluyvera ascorbata* as a significant pathogen in complicated UTIs are less common. Early recognition of this organism is crucial, especially in vulnerable patients with chronic illnesses or a history of multidrug-resistant infections. The identification of resistance patterns such as the AmpC phenotype observed in this isolate is highly significant, as it highlights the organism’s ability to harbor critical resistance mechanisms that limit therapeutic options and underscores the potential for underdiagnosis or underestimation of resistance in settings with limited microbiological capacity. This necessitates a tailored antibiotic approach based on culture and susceptibility testing results. Clinicians should be vigilant in monitoring such infections and be prepared to adjust treatment regimens promptly based on microbiological data and clinical response to mitigate the risk of severe complications [20,21,22].

### 3.3. Limitations

As a single-case study, the findings presented here may not be generalizable to all clinical settings. A significant limitation of this report is the absence of molecular testing for resistance genes. While phenotypic susceptibility testing, as performed using the MicroScan (Beckman Coulter, Brea, United States) and VITEK2 systems (bioMérieux, Marcy L’Étoile or Craponne, France) in accordance with CLSI guidelines, remains the standard for routine diagnostics, molecular methods (such as PCR or gene sequencing) would have provided definitive insight into the genetic basis of the observed AmpC resistance pattern and the presence of other resistance determinants. This would have allowed for a more precise characterization of the acquired resistance mechanism, which is particularly relevant for *K. ascorbate*, where AmpC is typically plasmid-mediated rather than chromosomal. Additionally, a limitation of this case report is the lack of repeated urine cultures during or shortly after the completion of antibiotic treatment, which would have provided formal microbiological confirmation of eradication. Despite these limitations, we believe the case remains clinically relevant and novel, particularly in the Colombian and Latin American context, where reports of *K. ascorbata* infections with this resistance pattern are rare. This case contributes to the awareness of this opportunistic pathogen in elderly patients with complex comorbidities, underscoring the need for further regional surveillance and microbiological capacity building, including access to advanced diagnostic techniques.

### 3.4. Future Recommendations

Further research is warranted to better characterize the clinical spectrum of *K. ascorbata* infections. Larger case series and controlled studies are needed to establish standardized treatment protocols and to assess the efficacy of alternative antimicrobial regimens. We recommend incorporating routine phenotypic screening for inducible AmpC production and molecular assays (such as PCR or gene sequencing) in microbiological workflows, especially in suspected multidrug-resistant isolates of *K. ascorbata*. Enhanced surveillance and multi-center collaborations are recommended to monitor emerging resistance trends and to develop evidence-based guidelines for managing infections caused by this emerging pathogen.

## 4. Conclusions

This case report underscores the clinical significance of *K. ascorbata* as an emerging pathogen in complicated urinary tract infections, particularly in vulnerable patients with chronic comorbidities such as chronic kidney disease and a history of multidrug-resistant infections. The identification of an *AmpC* resistance pattern in the isolate emphasizes the challenges posed by this organism, necessitating prompt and accurate microbiological diagnosis to guide effective antibiotic therapy.

The successful management of this case with meropenem highlights the importance of using broad-spectrum antibiotics in patients with a prior history of ESBL-positive infections. However, the presence of resistance genes such as *mcr-1* and *bla NDM-1* in similar isolates, as reported in the literature, raises concerns about potential treatment failures and the need for vigilant antimicrobial stewardship.

## Figures and Tables

**Table 1:** Laboratory analysis.

Test Name	Results	Reference Range
**Urinalysis**		
Specific gravity	1020	1.005–1.030
pH	2.25	7.32–7.45
Leukocyte esterase	+++	Negative
Blood	200	Negative
Bilirubin	Negative	0.1–1.2 mg/dL
**Sediment (Cells)**		
Bacteria	+++	Negative
Leukocytes	Uncountable	0–5 XC
RBCs (Erythrocytes)	40–60 XC	0–5 XC
Eumorphic	90%	–
Dysmorphic	10%	–
**Renal Function**		
Serum creatinine (SCR)	3.53 mg/dL	0.6–1.2 mg/dL
Blood urea nitrogen (BUN)	50.4 mg/dL	9–25 mg/dL
C-reactive protein (CRP)	38.4 mg/dL	<0.3 mg/dL
**Complete Blood Count (CBC)**		
WBC	15.11 × 10^3^/µL	4–10 × 10^3^/µL
Neutrophils (#)	12.58 × 10^3^/µL	1.8–6.5 × 10^3^/µL
Lymphocytes (#)	1.80 × 10^3^/µL	0.8–4.5 × 10^3^/µL
Monocytes (#)	0.50 × 10^3^/µL	0–1.2 × 10^3^/µL
Eosinophils (#)	0.19 × 10^3^/µL	0–1 × 10^3^/µL
Basophils (#)	0.04 × 10^3^/µL	0–0.3 × 10^3^/µL
Neutrophils (%)	83.2 × 10^3^/µL	45–65 × 10^3^/µL
Basophils (%)	0.3	0–3
Lymphocytes (%)	11.9	20–45
Monocytes (%)	3.3	0–12
Eosinophils (%)	1.3	0–10
Hemoglobin (Hb)	9.7 g/dL	13.8–17.2 g/dL
Hematocrit (HCT)	27.9%	40.7–50.3 %
MCV	95.2 fL	79–101 fL
MCH	33 pg	23–31 pg
RDW	14.6%	11–15 %
Platelets (PLT)	461 × 10^3^/µL	150–450 × 10^3^/µL

XC: examination count (cell count in sediment); SCR: serum creatinine; BUN: blood urea nitrogen; CRP: C-reactive protein; WBCs: white blood cells; HCT: hematocrit; MCV: mean corpuscular volume; MCH: mean corpuscular hemoglobin; RDW: red cell distribution width; PLTs: platelets; +++: Indicates a strong positive result or high presence (e.g., for leukocyte esterase and bacteria); (#): Indicates an absolute cell count (e.g., for white blood cell types).

**Table 2:** Antibiotic susceptibility profile of *K. ascorbata* in urine cultures.

**UROCULTURE A: Test Name: Results Uroculture A** **Method: MicroScan WalkAway ** **Organism: *Kluyvera ascorbata*** **Count: 100,000 CFU/mL**		
**Antibiotic**	**MIC**	**Interpretation**
Amikacin	≤8	S
Ampicillin	8	R
Ampicillin–Sulbactam	8-abr	S
Cefazolin	>8	R
Cefepime	≤1	S
Cefoxitin	>16	R
Ceftriaxone	≤1	S
Ciprofloxacin	≤0.125	S
Ertapenem	≤0.25	S
Gentamicin	≤2	S
Imipenem	0.5	S
Meropenem	≤0.5	S
Piperacillin–Tazobactam	≤4/4	S
Tigecycline	2	S
Trimethoprim–Sulfamethoxazole	≤0.5/9.5	S
**UROCULTURE B: Test name: Results** **Method: VITEK2 Compact system** **Organism: *Kluyvera ascorbata*** **Count: 100,000 CFU/mL**		
**Antibiotic**	**MIC**	**Interpretation**
Amikacin	≤2	S
Aztreonam	2	S
Ampicillin–Sulbactam	16-ene	I
Cefazolin	>64	R
Cefepime	≤1	S
Ceftazidime	≤1	S
Ceftriaxone	8	R
Ciprofloxacin	0.5	I
Ertapenem	≤0.5	S
Gentamicin	≤1	S
Meropenem	≤0.25	S
Piperacillin–Tazobactam	≤4	S
Trimethoprim–Sulfamethoxazole	≤20	S

**Table 3:** Comparison of relevant articles on *K. ascorbata*.

Study/Case Report	Main Pathology	Underlying Population/Conditions	Antimicrobial Resistance/Key Findings	Clinical Implications
Current Case	UTI	History of ESBL-positive *E. coli* UTI	Multidrug-resistant *K. ascorbata*, susceptible to fourth-generation cephalosporins.	Consider *K. ascorbata* in complicated UTIs, especially with resistance history.
Akiki et al., 2023 [1]	Septic shock	Urothelial cancer (immunocompromised)	*K. ascorbata* as a severe pathogen.	Severity in vulnerable populations, relevant to comorbidities.
Ulloa-Clavijo et al., 2023 [8]	Sepsis	Hemodialysis (chronic kidney disease)	*K. ascorbata* as a cause of sepsis.	Clinical suspicion in chronic kidney disease, relevant to potential comorbidities.
Álvarez and Ortiz 2021 [3]	UTI	Hospitalized patients	Resistance in urinary *Enterobacteriaceae*, including ESBL.	Supports the observation of resistance in the current case and ESBL history.
Janda et al., 2021, Liu et al., 2020 [5,6]	Not specified	Not specified	Update of taxonomic classification.	Importance of accurate identification to understand epidemiology and resistance.
Wajima et al., 2020 [7]	Not specified	Not specified	Identification of carbapenemases (IMP-1).	Justifies meropenem use due to ESBL history and potential resistance.
Sánchez. 2019 [4]	UTI	Not specified	Multidrug-resistant *K. ascorbata.*	Global relevance of *K. ascorbata* resistance and clinical impact.

## Data Availability

Data are contained within the article.

## Abbreviations

AMP	Ampoule
BUN	Blood urea nitrogen
CBC	Complete blood count
CFU	Colony forming unit
CKD	Chronic kidney disease
CMI	Minimum inhibitory concentration
CR	Creatinine
CRP	C-reactive protein
CTX-M-ase	Cefotaximase
ESBL	Extended-spectrum β-lactamase
HB	Hemoglobin
HCM	Mean corpuscular hemoglobin
HTO	Hematocrit
K	Potassium
MDR	Multidrug resistance
Na	Sodium
NSS	Normal solution saline
PLT	Platelet count
PUs	Pressure ulcers
R	Resistance
RDW	Red cell distribution width
S	Sensitive
SCR	Serum creatinine
UTI	Urinary tract infection
VCM	Mean corpuscular volume
WBC	White blood cell
